# Beliefs and Norms Associated with the Use of Ultra-Processed Commercial Milk Formulas for Pregnant Women in Vietnam

**DOI:** 10.3390/nu13114143

**Published:** 2021-11-19

**Authors:** Tuan T. Nguyen, Jennifer Cashin, Constance Ching, Phillip Baker, Hoang T. Tran, Amy Weissman, Thao T. Nguyen, Roger Mathisen

**Affiliations:** 1 Alive & Thrive Southeast Asia, FHI 360, Hanoi 11022, Vietnam; jcashin@fhi360.org (J.C.); cching@fhi360.org (C.C.); aweissman@fhi360.org (A.W.); rmathisen@fhi360.org (R.M.); 2 Institute for Physical Activity and Nutrition, Deakin University, Geelong, VIC 3220, Australia; phil.baker@deakin.edu.au; 3 Neonatal Unit and Human Milk Bank, Da Nang Hospital for Women and Children, Da Nang 50506, Vietnam; hoangtrandn@yahoo.com; 4 Asia Pacific Regional Office, FHI 360, Bangkok 10330, Thailand; 5 School of Biotechnology and Food Technology, Hanoi University of Science and Technology, Hanoi 11615, Vietnam; thao.nguyenthi@hust.edu.vn

**Keywords:** breastfeeding, breastmilk substitutes, code of marketing of breast-milk substitutes, commercial milk formula (CMF), conflict of interest, infant, nutrition, pregnant women, Vietnam

## Abstract

Commercial milk formula for pregnant women (CMF-PW) is an expensive, ultra-processed food with a high concentration of sugar, the consumption of which may be linked to negative health outcomes. However, CMF-PWs are promoted as beneficial for pregnant women and lactating mothers as well as their children. To date, little is known about the factors associated with the use of CMF-PW among pregnant women. We performed this analysis to examine the association between the use of CMF-PW and related beliefs and norms among pregnant women in Vietnam. We interviewed 268 pregnant women in their second and third trimesters from two provinces and one municipality representing diverse communities in Vietnam. Multinomial (polytomous) logistic regression, structural equation modeling (SEM), and propensity score matching (PSM) analysis were used to examine associations between beliefs and social norms related to CMF-PW and reported consumption, characterized as occasional, recent, and never during the current pregnancy. Overall, 64.6% of pregnant women reported using CMF-PW during the current pregnancy and 34.7% consumed CMF-PW on the day prior to the interview. Strong beliefs that CMF-PW will make a child smart and healthy (53.7%) and the perception that use of CMF-PW is common (70.9%) were associated with increased use on the previous day (beliefs: aOR: 3.56; 95% Confidence Interval (95% CI): 1.65, 7.71; *p* < 0.01 and social norms aOR: 2.29; 95% CI: 1.13, 4.66; *p* < 0.05). SEM and PSM analyses confirmed these findings for both occasional and regular CMF-PW use. Results are consistent with observations of CMF-PW product labels and marketing tactics in Vietnam. The prevalent use of CMF-PW in Vietnam is associated with the belief that these products make children smart and healthy and the perceived social norm that most mothers use these products, which mirrors marketing messages and approaches employed by the CMF industry.

## 1. Introduction

Pregnancy requires a healthy, balanced diet with sufficient energy, protein, and micronutrients obtained from diverse foods including vegetables, fruit, meat, fish, beans, nuts, and dairy [1]. According to the latest recommendations from the World Health Organization (WHO), all mothers should receive counseling on healthy eating and physical activity during pregnancy as well as daily iron and folic acid supplementation [1]. In contexts with a high prevalence of undernutrition and low consumption of nutrient dense foods, counseling to increase daily energy and protein intake is also recommended to reduce the risk of giving birth to a low birthweight baby [1]. Other interventions, including supplementation with calcium, Vitamin A, and intermittent iron and folic acid, are recommended in specific contexts. Pregnant women need to gain adequate weight to support fetal growth and development as well as prepare for the lactation after birth. The recommended weight gain depends on pre-pregnancy body mass index (BMI). Excessive weight gain during pregnancy is associated with negative health outcomes for both mother and child such as increased risk of gestational and pregestational diabetes, macrosomia, caesarean birth [2,3,4]. While once associated with wealth, overweight and obesity are now major public health problems in many low- and middle-income countries [5,6].

Commercial milk formulas for pregnant women (CMF-PW) are products marketed as nutritional supplements for pregnant and lactating women worldwide, primarily in middle- and lower-middle-income countries. The production, sale, and marketing of CMF-PWs are increasing globally, with the highest momentum in Asia, where an estimated 40% of new CMF-PW products were launched between 2013 and 2019 [7]. Websites of CMF-PW producers, distributors, and sellers indicate that CMF-PW are promoted through group meetings (e.g., prenatal classes), events, competitions, lucky draws, and one-on-one virtual consultations through social media [8]. These tactics are similar to those used to promote breastmilk substitutes (BMS), also referred to as CMF for infants and toddlers [9,10].

While the factors associated with the use of CMF for infants and toddlers and the impact of promotion were explored in the literature [9,10,11], little is known about the factors associated with the use of CMF-PW, despite the aggressive promotion of these products by the CMF industry and prevalent use among pregnant women. It is unclear whether these products are being ‘cross-promoted’ by using similar packaging and labelling as BMS for infants and young children. This study aims to address this gap by examining the association between the use of CMF-PW and related beliefs and social norms among pregnant women in Vietnam. To explore the links between pregnant women’s perceptions of CMF-PW and the messages used to market them, we also discuss the contents of CMF-PW labels and promotional materials in Vietnam.

## 2. Methods

### 2.1. Study Design, Participants, Sampling, and Data Collection

This posthoc analysis was based on a quantitative dataset of 268 pregnant women from a mixed method, cross-sectional study that collected primary data from 994 women of reproductive age, including pregnant women and mothers with infants aged 0–11 months in Vietnam [12]. The study was conducted in one province in the north, and one province and one municipality in the south of Vietnam. All data were collected in-person from May to July 2020. A more detailed description of the objectives, conceptual model, approaches, sampling, and sample size of that study can be found in the published research protocol [12].

We employed a stratified multiple-stage cluster sampling design [12,13]. Three provinces were selected purposively to represent diverse types of communities in Vietnam. Within each province, we listed all subdistricts under three categories: industrial zone, urban without an industrial zone, and rural without an industrial zone; and within each category, we randomly selected a district. Sampling within the selected districts was done in two stages stratified by province. Stage 1—selection of clusters/primary sampling unit (PSU): within each selected district, we listed all subdistricts and randomly selected 10 sub-districts. Stage 2—selection of participants: within each subdistrict, the research team selected pregnant women and mothers of infants aged 0–5 and 6–11 months using systematic random sampling from household lists provided by community health workers. Health workers then contacted selected women and invited them to participate. The nonresponse rate of this stage was 14.6%. Those who agreed to participate were contacted by the research coordinators who arranged for the interview [12,13]. The sample included both permanent and temporary residents (i.e., migrant workers) of the selected subdistricts. 

Data were collected electronically using tablets and uploaded daily to a secure cloud-based server and then reviewed by the data manager [13]. The data collection teams (including two supervisors and 18 enumerators) received training and were supervised by investigators at the Research and Training Centre for Community Development (RTCCD) and Alive & Thrive (A&T) [13]. Data were collected in accordance with the approved research protocol [12,13].

On average, enumerators took 45 min to complete an interview. To compensate participants for their time, we provided VND 100,000, equivalent to $4.50 (US Dollars), to at the end of each interview [13]. Interviewees had the option to select a gift such as a raincoat or a parenting book in lieu of the cash gift.

### 2.2. Variables

#### 2.2.1. Outcome Variables

The main outcome variable for this analysis was CMF-PW use, based on women’s answer to two questions: “During this pregnancy, did you drink milk for pregnant women?” and “Did you drink milk for pregnant women yesterday?” Respondents were then grouped into one of three mutually exclusive categories based on their responses: (1) did not use CMF-PW (nonusers); (2) used CMF-PW at least once during the pregnancy but not on the previous day (occasional users); and (3) used CMF-PW yesterday (recent users).

#### 2.2.2. Exposure Variables

To measure respondents’ beliefs related to CMF-PW, we asked pregnant women to indicate, using a Likert scale of 1 (strongly disagree), 2 (disagree), 3 (somewhat disagree), 4 (somewhat agree), 5 (agree), and 6 (strongly agree), their agreement with the statement: “If I drink milk for pregnant women, my newborn will be smart and healthy.” We then dichotomized the variable to “Agree” (score of 5 or 6) and “Not agree” (scores 1 to 4).

To measure perceived social norms related to CMF-PW, we asked women to indicate their agreement with the statement: “Most of the pregnant women I know consume(d) milk for pregnant women” using the same Likert scale. The variable was then dichotomized to “Agree” (score of 5 or 6) and “Not agree” (scores 1 to 4).

#### 2.2.3. Covariates

A breastfeeding knowledge score (range of 0–3) was calculated as the sum of correct answers to three questions related to recommended timing of early (<1 h), exclusive (6 months), and continued breastfeeding (18–36 months). The use of at least 18 months was based on national guidelines [14]. We also asked about women’s exposure to promotion of CMF-PW, including advice or recommendation, free samples, coupons, or gifts in the previous 30 days.

For 158 women, we estimated gestational age based on the interview date and last reported menstrual period. For women whose last menstrual period date was unknown (*n* = 110), we estimated gestational age based on maternal recall of the information provided during antenatal care (ANC). We also asked respondents how many children they had under the age of five.

We collected data on the location(s) of ANC, which was then categorized into: public health facilities only, both public and private health facilities, and private health facilities only. We asked how many ANC visits (continuous) a woman attended up to the time of interview and whether she had been approached by a CMF industry representative during an ANC visit at a health facility during this pregnancy. The number of ANC visits, which is dependent on gestational age, is a proxy for contacts with health workers and potential opportunities for breastfeeding and maternal nutrition counseling and/or CMF promotion.

We collected socioeconomic characteristics of participating pregnant women, including, age (years), ethnicity, marital status (married or unmarried), living with a partner, education (primary school or less, junior secondary school, secondary school, diploma or postgraduate), and employment status (blue-collar and farmer, white-collar, small trader or self-employed, and unemployed, homemaker, student, or other).

### 2.3. Data Analysis

Data analysis was performed using Stata 15.1 (StataCorp LLC, College Station, TX, USA). For regression models, we adjusted for the clustering (e.g., province or municipality and the 30 PSUs within each province) by using the robust option. We neither estimated sampling weights nor used them in the analysis because our primary focus was on the assessment of association rather than the estimation of prevalence. Three different regression models were used: multinomial (polytomous) logistic regression, structural equation modeling (SEM), and propensity score matching (PSM). We used full regression models for these analyses and did not use any cut points to exclude any variables. While each of these approaches have methodological limitations, together they can provide convincing evidence for the association if they support consistent conclusions.

Multinomial (polytomous) logistic regression: we performed this analysis to examine associations between beliefs and social norms relating to CMF-PW and the use of CMF-PW during this pregnancy. The use of CMF-PW was defined by the three mutually exclusive categories described above. This approach explored whether there was an association between beliefs and norms and the use of CMF-PW, controlling potential confounding factors, but ignoring potential pathways of association. We partially addressed this limitation by conducting a SEM analysis.

SEM: we conducted a generalized SEM analysis using the *gsem* command in Stata to examine associations between beliefs and social norms and the use of CMF-PW (occasional and regular use versus never using) in relation to other covariates, guided by a conceptual model described in our study protocol [12,13]. We used a multinomial model with a logit link. In this analysis, beliefs, social norms, and breastfeeding knowledge are intermediate outcomes also predicted by other covariates. A limitation of both multinomial logistic regression and SEM is the lack of an explicit control group. We partially addressed this limitation by conducting a PSM analysis [15].

PSM: PSM minimizes the effect of differential information bias related to the beliefs and norms themselves (e.g., recall of using CMF-PW) and/or unmeasured variables that are associated with the outcomes and exposures. For each of the exposure variables, we used command *teffects psmatch* in Stata to create groups of pregnant women exposed and unexposed to beliefs or social norms based on similarity of estimated propensity scores. The selection of the matched group was based on 1: 1 nearest neighbor matching within a caliper [15]. The propensity scores were estimated using a logit model based on various characteristics of the women (age, ethnicity, education, job, living with partners, had a child aged five years or younger, province or municipality of residency), breastfeeding knowledge, gestational age, number of ANC visits, and location of ANC visits.

## 3. Results

In the sample of 268 pregnant women, 93.3% belonged to the majority Kinh ethnicity, 99.3% were married, 96.3% were currently living with partners/husbands, 21.6% had a white-collar job, and 58.2% had ≥12 years of education. The mean age of the women was 29.3 years (Table 1).

The proportion of pregnant women who used CMF-PW at some time during the current pregnancy was 64.6% and on the previous day was 34.7% (Table 2). More than half (53.7%) of pregnant women believed or strongly believed that CMF-PW would make a child smart and healthy, and 70.9% agreed or strongly agreed that most of the pregnant women they knew consumed CMF-PW (Table 2). A quarter of the pregnant women were exposed to a promotion for CMF-PW in the previous 30 days and 29.1% correctly answered three questions on recommended timing for early, exclusive, and continued breastfeeding (Table 2).

Participants had a mean gestational age of 25.7 weeks, with 43.7% in the third trimester of pregnancy (Table 3). The average number of ANC visits was four. Private health facilities were more commonly visited for ANC, with 51.5% visiting private facilities only and 21.3% visiting public facilities only (Table 3). Despite national regulations, 18.3% of pregnant women reported being approached by a CMF industry representative who promoted CMF or collected personal information from them during their ANC visits (Table 3).

In the multinomial (polytomous) logistic regression model, beliefs (aOR: 3.56; 95% CI: 1.65, 7.71; *p* < 0.01) and social norms (aOR: 2.29; 95% CI: 1.13, 4.66; *p* < 0.05) relating to CMF-PW were significantly associated with recent consumption of CMF-PW (on the previous day) (Table 4). Similarly, beliefs (aOR: 1.94; 95% CI: 0.90, 4.20; *p* = 0.090) and social norms (aOR: 1.69; 95% CI: 0.76, 3.78; *p* = 0.196) were associated with occasional consumption of CMF-PW during the pregnancy (Table 4). Further, having more ANC visits was associated with increased likelihood of using CMF-PW sometime during the pregnancy (Table 4).

**Figure 1:**
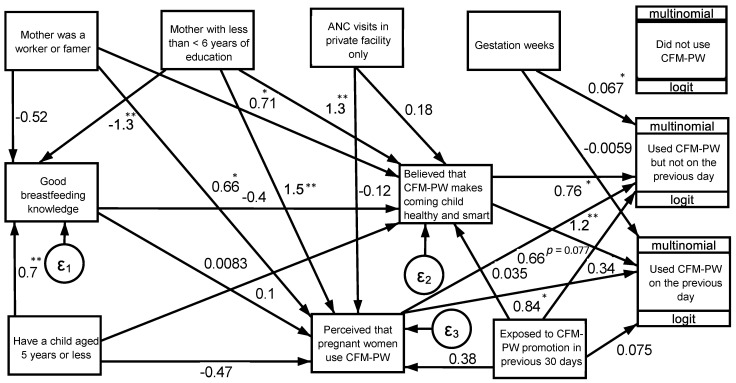
Structural equation modeling (SEM) analysis for association among beliefs and social norms and use of commercial milk for pregnant women (CMF-PW). Boxes indicate measured variables; arrows indicate direction of association; and values are coefficient, *n* = 268. * *p* < 0.05, ** *p* < 0.01, other *p* < 0.10 were indicated by exact value.

SEM analysis showed that a respondent’s belief that CMF-PW will make a child smart and healthy was positively associated with recent (β: 1.17; *p* < 0.01) and occasional CMF-PW consumption (β: 0.76; *p* < 0.05) (Figure 1). The perception (social norm) that most pregnant women use CMF-PW was also associated with recent (β: 0.84; *p* < 0.05) and occasional CMF-PW use β: 0.66; *p* = 0.077) (Figure 1). A higher gestational age was associated with increased likelihood of consuming CMF-PW on the previous day (β: 0.67; *p* < 0.05). These beliefs and norms were associated with lower maternal education (elementary school or less vs. other) and being a blue-collar employee (Figure 1).

The PSM analysis confirmed these relationships. The belief that consumption of CMF-PW will make a child smart and healthy was associated with an increased likelihood of using CMF-PW in the pregnancy (0.27 percentage points; 95% CI: 0.15, 0.39; *p* < 0.001) and on the previous day (0.29 percentage points; 95% CI: 0.17, 0.42; *p* < 0.001) (Data not shown). The perception that most pregnant women use CMF-PW was also associated with increased likelihood of using CMF-PW in the pregnancy (0.18 percentage points; 95% CI: 0.05, 0.31; *p* < 0.01), but not on the previous day (0.11 percentage points; 95% CI: −0.07, 0.29; *p* = 0.23) (data not shown).

## 4. Discussion

The aim of this study was to examine the association between the use of CMF-PW among expectant mothers in Vietnam and their beliefs and perceived social norms related to these products. We found that CMF-PWs are widely used by pregnant women in Vietnam; different regression models consistently show that the use of these products is strongly associated with the belief that these products are beneficial for the mother and/or the child, and the perception that other pregnant women use CMF-PW. This association is strongest among recent users of CMF-PW.

These findings are unsurprising when we consider the aggressive tactics used to market CMF in Vietnam. In the sections that follow, we describe this context, including an analysis of CMF-PW promotional messages and labeling and evidence of cross-promotion of CMF-PW with other products; industry presence in health facilities; and interactions with government on research and guideline development.

### 4.1. Promotional Messaging and Claims about the Benefits of CMF-PW 

**Figure 2:**
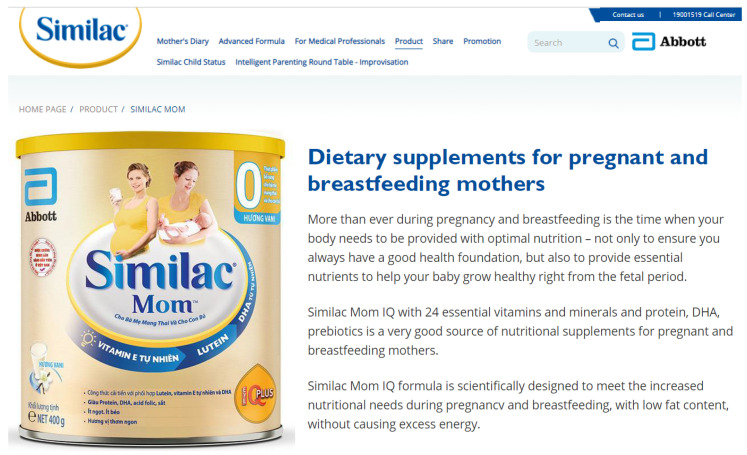
Benefit icons and nutrition and health claims on label of Similac Mom [8]. Picture of baby indirectly confers that these benefits extend to baby too.

The belief that using CMF-PW during pregnancy will make a child smart and healthy is aligned with the promotional messaging and claims of these products (Figure 2), which appeal to parents’ emotions and aspirations, including child learning ability, brain development, and scholastic achievement [10,16]. “Nutritional positioning”, a technique involving the development of products with novel ingredients and implied or direct claims on product labels, including those relating to brain, eye, and immune system development, among others, are used to reinforce these beliefs [17,18]. Similar claims are commonly used in the marketing of CMFs for infants and toddlers [10,11].

The use of these tactics is prominent in Vietnam, where the most popular CMF-PW brands [19] utilize health and nutrition claims for pregnant women, the fetus, and the child both on product labels and in promotions on several media platforms, including digital. CMF-PW labels highlight specific ingredients and their biological functions, which are used as nutrition and health claims for the child, pregnant woman, and mother. For instance, the icon of an “Eye-Q Plus” droplet on the label of Similac Mom Eye-Q (Image 1) conveys the message that the ingredients (e.g., vitamin E, lutein, and DHA (Docosahexaenoic acid)) can enhance vision (eye) and intelligence (IQ). The picture of a mother holding an infant indirectly communicates that the product confers such benefits to the child through the mother’s consumption. The label also contains a statement, “the first [product] approved clinically in Vietnam [that] helps to meet recommended head circumference”, which is a claim referring to infant brain development (translated from Vietnamese). On the Similac Vietnam website [8], the claims directly refer to the baby, stating the product “helps with brain development”, and “contains a unique IQ nutrient system including DHA, choline, folic acid and iron—essential nutrients for the baby’s brain”.

**Figure 3:**
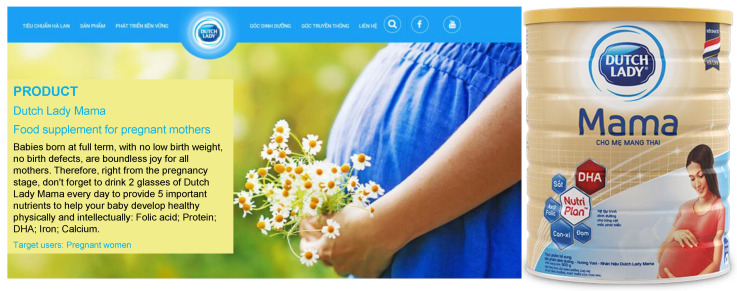
Dutch Lady Mama’s benefit icons convey several health claims for babies [20].

**Figure 4:**
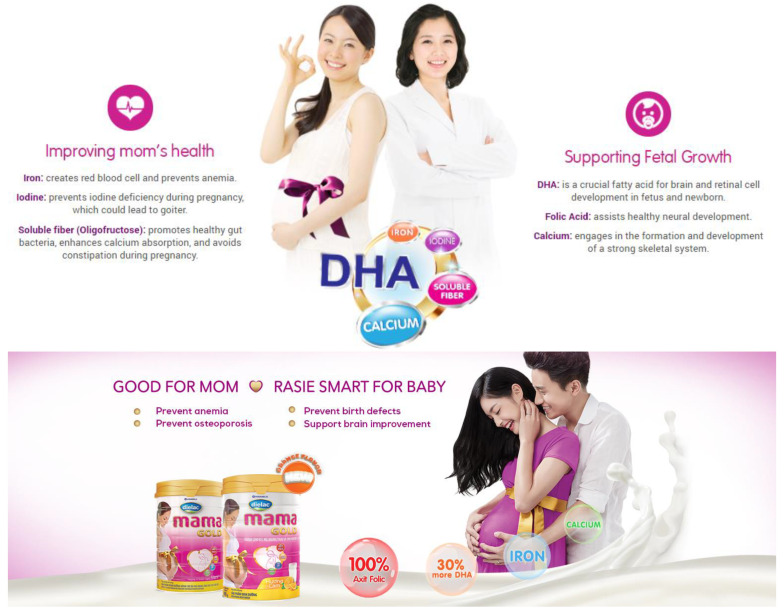
Dielac Mama Gold claims product benefits health of mother, fetus, and newborn [21].

**Figure 5:**
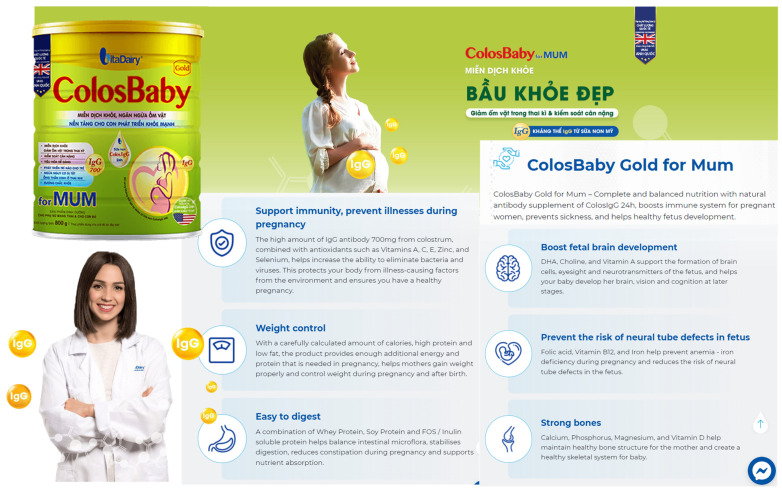
ColosBaby for Mum claims product benefits health of mother, fetus, and newborn [22].

Similar claims are used by Dutch Lady Mama [20], which states on their website “five important nutrients to help your baby develop healthily physically and intellectually” (Figure 3). Another brand, Dielac from Vinamilk [21], uses the slogans “Healthy Moms, Smart Babies” and “Good for Mom, Raise Smart for Baby [Raise Smart Baby]” on their website (Figure 4), with various health claims such as prevention of anemia, osteoporosis, birth defects, and supporting fetal brain development [21]. Promotional materials for ColosBaby for Mum highlight added contents such as milk from cows that have just given birth to claim that the product will “Bolster immunity” in mothers (Figure 5) [22]. These claims align with this study’s findings that many pregnant women believe that consumption of CMF-PW contributes to smart and healthy babies.

In Vietnam, the Code prohibits the use of images of infants or fetuses on advertisements for CMF-PW [23]. In violation of national law, the label for Abbott Similac Mom Eye-Q includes a picture of an infant (Image 1) [8] and VitaDairy label uses a heart shape on the mother’s belly that refers to the fetus on the ColosBaby for Mum (Image 4). Promotional materials for the product feature an image of a person in a white coat that suggests that the product is endorsed by health professionals (Figure 3 and Figure 4) [22].

### 4.2. CMF Market Segmentation and Cross-Promotion Strategies 

The promotion of CMF-PW products reflects a market segmentation and cross-promotion strategy, whereby product ranges extend from "womb-to-tomb,” including CMFs not only for pregnant women and lactating mothers, but also for infants, children, adolescents, and the elderly [9].

Prior to the adoption of the Code in 1981, infant formula was the main product promoted by the CMF industry from birth, without an upper-age limit. However, as infant formula marketing regulations tightened after the adoption of the Code, the industry expanded its product range to include products marketed for older infants and young children to expand markets and sustain sales. The promotion of CMF-PW is another extension of this strategy. Furthermore, by using similar branding and packaging across CMF products, including infant formula, the various categories are cross promoted, even in countries where such promotion is prohibited by law [11,24,25].

**Figure 6:**
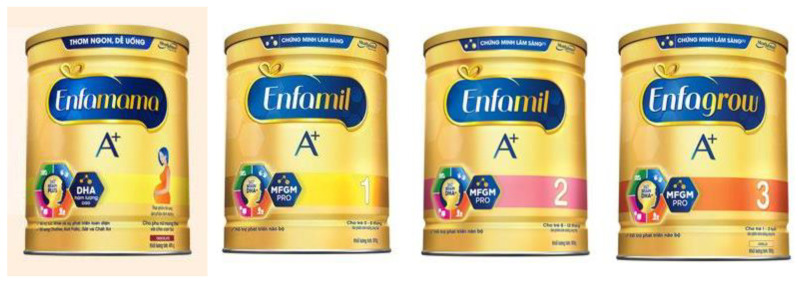
Cross-promotion of products in Enfa product line, with almost identical packaging and labeling [27].

**Figure 7:**
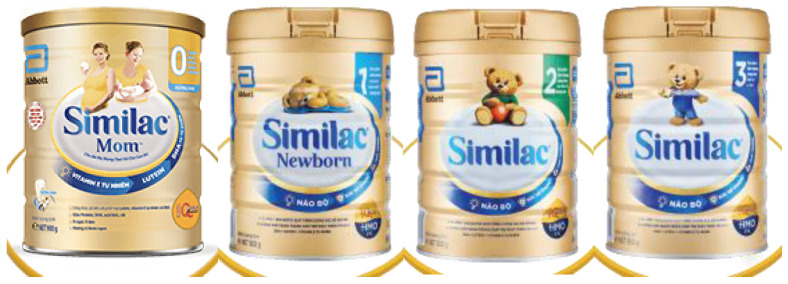
Cross-promotion of products in Similac Eye-Q product line, with almost identical packaging and labeling [8].

**Figure 8:**
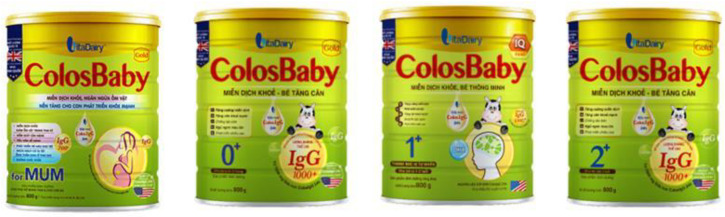
Cross-promotion of products in ColosBaby product line, with almost identical packaging and labeling [22].

The cross-promotion of CMF-PW with other types of CMF within the brand can be readily observed in Vietnam [26]. For example, CMF-PW packaging design, graphics, and color schemes are almost identical across the product line, as illustrated by Enfamama (Figure 6) [27], Similac Mom Eye-Q (Figure 7) [8], and ColosBaby (Figure 8) [22]. Messaging and symbols are also consistent across products (“360 brain DHA+” and “DHA/MFGM PRO” for Enfa products; the Eye-Q icons for Similac products), serving to promote multiple claims and to distinguish the range of branded products from others. 

Cross-promotion not only creates brand identity (and presumed brand loyalty), but it also confuses consumers. For example, follow-up and toddler milks are frequently mistaken by parents and caregivers for standard infant formula [24]. Studies in Australia and Italy found that 67% and 81% of mothers, respectively, reported seeing an infant formula advertisement when in fact they saw an advertisement for a toddler milk formula, which is not prohibited by local law [25,28]. Cross-promotion of BMS with other CMFs is thus potentially dangerous, undermines breastfeeding, and violates the Code [29].

### 4.3. The CMF Industry Promotes CMF-PW with Approaches Prohibited under the Code

The CMF industry promotes CMF-PW in health facilities, provides free samples, sponsors research, and engages in national policy development, all in violation of the Code. In our study, ANC visits were positively associated with occasional CMF-PW use, but not recent consumption. These findings may be explained by the widespread practice of providing free samples of CMF-PW to pregnant women either during ANC, prenatal classes or events, calls, or through online communication and other promotional activities (e.g., lucky draw), which encourage pregnant women to try CMF-PW [8]. In-depth interviews with these same women, described in another paper [13], revealed that CMF industry representatives approached them during antenatal visits to collect their contact information, expected date of birth, and other personal information to facilitate online and in-person CMF promotion. The study also showed that in the 30 days preceding the survey, 28.0% of pregnant women were exposed to promotion of CMFs (either CMF-PW or BMS), 23.9% to promotion of CMF-PW, and 8.6% to promotion of BMS [13].

### 4.4. The CMF Industry Sponsors Research and Influences Health Policy

In Vietnam, CMF producers sponsored studies on CMF-PW led by authors from the CMF industry (Abbott Nutrition), in collaboration with the government and research institutions. These studies, which have inherent conflicts of interest, reported a positive association between the use of a CMF-PW product sold by the company that sponsored the research (Similac Mom Eye-Q) and birth outcomes, child development, and successful breastfeeding [30,31,32]. Using the findings from these studies, the CMF industry influenced the development of the “National Guideline on Nutrition for Pregnant Women and Lactating Mothers” (the National Guideline), launched by the Vietnamese Ministry of Health in 2017 through technical and financial support from Abbott Laboratories [33]. The National Guideline recommends CMF-PW over other milk and dairy products by emphasizing that CMF-PW is “scientifically produced to meet increasing nutritional demands [energy, nutrients] during pregnancy and lactation periods” and that “[women] should choose clinically proven products” [33].

By influencing research and policy through sponsorship, CMF-PW is also indirectly promoted as a product group to create a generic marketing effect. It attempts to change the narrative about CMF consumption by kickstarting it at pregnancy. Having the country’s highest health authority officially endorse these products in a public health policy instrument confers a seal of approval to the public [23,34]. Such endorsement is also likely to trickle down to the health system, gaining goodwill with healthcare workers that can result in promotion of these products to pregnant women and lactating mothers [35]. In addition, the CMF industry sponsors health professionals to participate in training courses as well as in national and international conferences and provides financial support to pediatric and obstetric health centers for different activities [36]. These opportunities present a conflict of interest by leading health professionals to become promoters of CMF products.

### 4.5. CMF-PW Promotion Should Be Better Regulated

Nutrition guidelines for women during pregnancy and lactation from WHO [37] and various countries, including the US [38], UK (United Kingdom) [39], Australia [40], as well as those reported in systematic reviews of studies in North America, Europe, and East and South Asia [41,42] recommend balanced diets for women during pregnancy and lactation and do not provide any specific recommendations related to CMF-PW. Indeed, most guidelines encourage consumption of low-fat dairy products and multi-nutrient supplements to avoid excessive weight gain during pregnancy. Systematic reviews showed positive associations between low-to-moderate maternal milk intake compared to no or limited intake of dairy products during pregnancy for both infant birthweight and length [43,44].

A randomized, controlled population study in Vietnam led by Abbott to test their product (Similac Mum; Abbott Laboratories, Vietnam) showed positive impact of CMF-PW supplementation along with counseling on birthweight and exclusive breastfeeding compared to those who received the local standards of care: ANC visits, breastfeeding counseling if available, and folic acid (400 mcg) and iron (60 mg) supplement [31]. The study was based on a small sample size (*n* = 228 at randomization and *n* = 204 at the completion of the study) of first-time mothers with pre-pregnancy body mass index of <25 kg/m^2^. The higher exclusive breastfeeding prevalence could be due to their regular counseling along with the supplementation [31]. Also, the authors did not present the mean birthweight, thus, the increased birthweight could be hazardous rather than beneficial [31]. Based heavily on this study, the National Guidelines from Vietnam (developed and sponsored by Abbott) are among few guidelines to explicitly recommend CMF-PW [33].

In addition to overemphasizing the benefit of CMF-PW, CMF producers withhold information about potential harms related to the use of CMF-PW—an ultra-processed food with a high concentration of added sugar [45,46,47,48]. Like other CMFs, CMF-PW products contain no whole foods, are manufactured through a series of industrial processes, and contain cosmetic additives [49]. Specifically, the production of CMF products, including CMF-PW, involves a two-stage process, which involves denaturing and changing the composition of proteins, other macronutrients, micronutrients, trace elements, enzymes, and bioactive substances, and then adding some of the nutrients back [48].

CMF-PW also has an elevated level of added sugar and energy. Although it is not easy to tell the exact amount of added sugar because the CMF industry reports the amount of carbohydrates rather than sugar, given the production procedure, one could expect that most of the carbohydrates in CMF-PW come from added sugar [45,46,48]. Indeed, sugar is among the top two ingredients of CMF-PW, just after milk powder [8,20,27,34]. Compared with 100 mL of mature breastmilk (6.7 g of natural lactose, 65–70 Kcal) [50] and pasteurized whole cow milk (5 g of natural lactose, 70 Kcal) [51], CMF-PW brands have a much higher concentration of sugar (assuming all carbohydrates are from sugar) and energy: Abbott’s Similac Mom (11 g of sugar, 71 Kcal) [8]; Mead Johnson’s Enfamama A+ (15 g of sugar, 75 Kcal) [27]; Vinamilk’s Dielac Mama Gold (13 g of sugar, 100 Kcal) [21]. The sugar content in these CMF-PWs is higher than in 100 mL of Classic Coke (10.6 g of sugar, 45 Kcal) [52]. Dietary guidelines throughout the world, including Vietnam, recommend limiting consumption of added sugar, especially for pregnant women [33,53,54,55]. High intake of energy and sugar and consumption of ultra-processed foods is linked with excessive weight gain during pregnancy and increased risk of gestational and pregestational diabetes, macrosomia, caesarean birth, and adverse health outcomes for both mother and child [2,3,4,56].

### 4.6. Study Strengths and Limitations

To our knowledge, this is the first study to examine the beliefs and norms associated with the use of CMF-PW and to analyze the marketing context that influences them. However, our study has several limitations, including cross-sectional design with descriptive data analysis. Because this design provides a snapshot of the situation in purposively selected provinces of Vietnam, the findings cannot be generalized to Vietnam as a whole or to other countries. However, given that the CMF market leaders in Vietnam are multinational companies, it is likely that the marketing tactics used in Vietnam are also applied in other countries and regions. Another limitation is the nonresponse rate of our sampled population. In-person data were collected during the COVID-19 pandemic, which made some women hesitant to participate in the interview (non-response rate of 14.6% in the initial enquiry for the meeting). Relatedly, restrictions on group activities, events, and access to health facilities during the data collection period possibly led to an underestimation of the interpersonal, face-to-face promotion of CMF products in the findings and an overestimation of promotion on digital platforms.

Further, because this study was a posthoc analysis of data collected for another purpose (i.e., associated factors of breastfeeding practices and the use of BMS), the sample size was not determined for this study’s purpose. However, the sample size was sufficient to estimate our models and assess the association of interest. Finally, because it was not the purpose of the main study, we cannot provide a complete picture of all the factors associated with the use of CMF-PW, perceptions of promotional materials and labels used to market these products, nor of the impact of CMF-PW use. For example, we were not able to explore other beliefs concerning CMF-PW’s perceived effect on immunity, prevention of illness during pregnancy, weight control, constipation prevention, improved sleep, and improved eye health. Further study on this topic is needed.

## 5. Conclusions

We found that the prevalent use of CMF-PW in Vietnam is associated with the belief that these products make children smart and healthy and the perceived social norm that most pregnant women use these products. The study findings suggest that the CMF industry uses CMF-PW as an entry point to further promote other CMF products, including BMS, in multiple settings (e.g., health facilities, shops, pharmacies, perinatal classes or events, online forums, advertisement), directly to women, other caregivers, and the public, both in-person and online. They also influence CMF-PW norms through sponsorship of health worker training, research, and even national policy development. Misleading nutrition and health claims and ambiguous information (e.g., carbohydrate vs. sugar content) not only convinced women that the product is beneficial for their own health and for the health of their babies, without considering potential harms, but also contributed to brand affinity with the goal of continued CMF use after childbirth, undermining breastfeeding.

Even though there are provisions in the Vietnamese Code (100/2014/ND-CP) that place restrictions on how CMF-PW can be advertised, regulations on the promotion of CMF-PW need to be strengthened, especially at health facilities, in public, on social media, and through sponsorship from CMF industry. There is also an urgent need for stronger regulation on the labeling of CMF-PW, including the use of images, nutrition and health claims, cross-promotion with CMF for infants and toddlers, and the sugar content of these products. Finally, the potential harmful effects of using CMF-PW must be communicated to pregnant women, mothers, and the health workers that counsel them.

## Figures and Tables

**Table 1:** Socioeconomic characteristics of pregnant women ^1^.

	Estimates (*n* = 268)
Kinh ethnicity	93.3
Age (Mean ± SD; Median (*p*25–*p*75))	29.3 ± 5.9 29 (25–33)
Married	99.3
Living with partners	96.3
Highest level of education:	
Primary school or less	17.5
Junior secondary school	24.3
Secondary school	23.1
Some college or higher	35.1
Main occupations:	
Blue-collar or farmer	19.4
White-collar	21.6
Small trader, self-employed, small self-owned business, services	34
Unemployed, homemaker, student	25

^1^ Values are % or, when specified, mean ± SD, median, (*p*25–*p*75).

**Table 2:** Use of commercial milk formula for pregnant women (CMF-PW), exposure to promotion of CMF-PW and breastfeeding knowledge ^1^.

	Estimates (*n* = 268)
Use of CMF-PW:	
Have not used	35.4
Have used, but not on the previous day	29.9
Used on the previous day	34.7
Believed that CMF-PW makes a child smart and healthy ^2^	53.7
Perceived that most pregnant women use CMF-PW ^2^	70.9
Exposed to promotion of CMF-PW in the previous 30 days	23.9
Ever contacted by CMF industry representative during ANC visit	18.3
Breastfeeding knowledge:	
Breastfeeding knowledge score ^3^ (Mean ± SD; Median (*p*25–*p*75))	1.9 ± 0.9 2 (1–3)
No correct responses	4.9
Correct response to 1 question	25.4
Correct response to 2 questions	40.7
Correct response to all 3 questions	29.1

^1^ Values are % or, when specified, mean ± SD, median (*p*25–*p*75). ^2^ Level of agreement of 5 (agree) or 6 (strongly agree) from a Likert scale of 1–6. ^3^ Score (ranged 0–3) was sum of correct answers to 3 questions relating to timing of early, exclusive, and continued breastfeeding.

**Table 3:** Proportion of respondents with children under 5 years, frequency and location of antenatal care, and gestational age ^1^.

	Estimates (*n* = 268)
Location of ANC:	
Public health facilities only	21.3
Both public and private health facilities	27.2
Private health facilities only	51.5
Gestation weeks (Mean ± SD; Median (*p*25–*p*75))	25.7 ± 7.6 26.8 (20.9–32.0)
In 3rd trimester	43.7
Number of ANC visits (Mean ± SD; Median (*p*25–*p*75))	4.2 ± 3.4 3.5 (2–6)
Had a child aged 5 years or less	44.0

^1^ Values are % or, when specified, mean ± SD, median (*p*25–*p*75).

**Table 4:** Adjusted odds ratios (aOR) and 95% confidence intervals (95% CI) for associated factors of using commercial milk formula for pregnant women (CMF-PW, *n* = 268) ^1^.

	Occasional Consumption	Recent Consumption
Believed that CMF-PW makes a child smart and healthy ^2^	1.94 (0.9, 4.2)	3.56 ** (1.65, 7.71)
Perceived that most pregnant women use CMF-PW ^2^	1.69 (0.76, 3.78)	2.29 * (1.13, 4.66)
Breastfeeding knowledge score ^3^	1.17 (0.74, 1.85)	1.22 (0.82, 1.83)
Exposed to promotion of CMF-PW in the previous 30 days	1.77 (0.77, 4.07)	0.99 (0.45, 2.17)
Location of ANC:		
Public health facilities	1 (1, 1)	1 (1, 1)
Both public and private health facilities	1.17 (0.42, 3.24)	1.28 (0.52, 3.13)
Private health facilities	1.43 (0.59, 3.51)	0.84 (0.42, 1.67)
Ever contacted by CMF industry representative during ANC visit	1.2 (0.49, 2.93)	2.02 (0.82, 4.98)
Gestation weeks	0.91 (0.81, 1.02)	0.99 (0.89, 1.09)
Number of ANC visits	1.09 ** (1.03, 1.16)	0.99 (0.95, 1.03)
Had a child aged 5 years or less	0.54 (0.28, 1.03)	0.85 (0.42, 1.7)

^1^ Data from The Code impact study in Vietnam in 2020. Values are adjusted odds ratios (aOR) and 95% confidence intervals (95% CI) from multinomial (polytomous) logistic regression, controlled for maternal ethnicity, education, job, living with partners. We used robust option to account for clustering. Significantly different from null value (aOR = 1; two-sided t tests): * *p* < 0.05, ** *p* < 0.01. ^2^ Level of agreement of 5 (agree) or 6 (strongly agree) from a Likert scale of 1–6. ^3^ Score (ranged 0–3) was sum of correct answers to 3 questions relating to timing of early, exclusive, and continued breastfeeding.

## Data Availability

Requests for data may be directed to the corresponding author and are subject to institutional data use agreements.
